# Three‐reaction high‐resolution melting assay for rapid differentiation of *Mycobacterium tuberculosis* complex members

**DOI:** 10.1002/mbo3.919

**Published:** 2019-08-25

**Authors:** Patricia Landolt, Roger Stephan, Marc J. A. Stevens, Simone Scherrer

**Affiliations:** ^1^ Section of Veterinary Bacteriology, Institute for Food Safety and Hygiene, Vetsuisse Faculty University of Zurich Zurich Switzerland

## Abstract

The possibility of introducing a reliable assay for a quick identification and differentiation of the main species of *Mycobacterium tuberculosis* complex (MTBC) supports the improvement of efficient tuberculosis combating strategies worldwide. Commercially available assays are often based on cultured samples; however, due to the long cultivation time of mycobacteria, results are delayed. Developed PCR approaches have been published previously, though, when testing intricate veterinary samples, the complex composition of multiplex qPCRs frequently leads to assay failure. In order to overcome those limits, a paradigm of a three‐reaction high‐resolution melting (HRM) assay for the simultaneous identification and differentiation of the main members of MTBC was established. The assay is based on single nucleotide polymorphisms within *gyrB* and *gyrA*, which have been used as target for the establishment of two highly specific HRM assays (HRM assays 1 and 2) discriminating *M. tuberculosis*/* Mycobacterium canetti*,* Mycobacterium bovis*/*M. bovis* BCG,* Mycobacterium caprae*/rare *M. caprae*/*M. bovis* ecotypes,* Mycobacterium africanum*/*Mycobacterium orygis*/* Mycobacterium pinnipedii*/Clade A1,* Mycobacterium microti*, and a rare subtype of *M. canettii* followed by a third HRM assay (HRM assay 3) allowing a further differentiation of *M. bovis*, *M. bovis* BCG, and a rare subtype of *M. caprae*/*M. bovis*, which is considered to be a novel ecotype. High‐resolution melting assay 1 is described in a previously published report. High‐resolution melting assay 2 showed 100% correlation of all 39 examined isolates with the results of a commercial identification kit. 96% of the clinical samples tested demonstrated concordant results. High‐resolution melting assay 3 showed an accordance of 100% with the results of the commercially available identification kit of all 22 samples analyzed. The proposed strategy of the three‐reaction HRM assay can be used for an accurate differentiation of up to seven groups of MTBC and potentially to identify a rare subtype of *M. canettii* either on isolates or on clinical samples.

## Graphical abstract

The HRM assay enables rapid identification and differentiation of the clinically most relevant species of MTBC namely *M. tuberculosis*, *M. africanum*, *M. microti*, *M. bovis*, *M. caprae*, and *M. bovis* BCG from directly extracted samples and cultured material. The use of this powerful assay may save several months of cultivation time and supports improvement of efficient tuberculosis combating strategies worldwide.

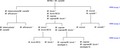

## 1 INTRODUCTION

Tuberculosis is a major cause of human death induced by only one infectious agent resulting in approximately 10 million new infections per year along with about 1.6 million deaths in 2017 (WHO, 2018). Tuberculosis persists as a major health concern not only in humans but also in veterinary medicine.

*Mycobacterium tuberculosis* complex (MTBC) comprises the closely related species *Mycobacterium (M.) tuberculosis*, *M. bovis*, *M. bovis* Bacillus Calmette and Guérin (BCG), *M. caprae*, *M. africanum*, *M. microti*, *M. pinnipedii*,* M. orygis*, and four further species known as animal‐adapted Clade A1 (Dassie bacillus, *M. mungi*, Chimpanzee bacillus, and *M. suricattae*) (Brites et al., 2018). Furthermore, *Mycobacterium canettii* is a genetically more diverse and recombinogenic organism as observed earlier (Fabre et al., 2010), only leading to opportunistic human infections from time to time (Boritsch et al., 2016; Supply et al., 2013). Although its similarity of the nucleotide codes to the species of MBTC, it is not considered to be part of MTBC (Brites et al., 2018). *Mycobacterium canettii* is mainly limited to the horn of Africa and most of the known strains were isolated in the Republic of Djibouti (Blouin et al., 2014). *Mycobacterium tuberculosis* is known to be the major source of human tuberculosis; however, numerous cases of infection with other members of the complex are known. *Mycobacterium bovis* and more rarely *M. caprae* are the causative agents for bovine tuberculosis, which is recognized to be an important zoonosis responsible for significant economic loss (Rodriguez‐Campos, Smith, Boniotti, & Aranaz, 2014). A recent study (Loiseau et al., 2019) revealed two rare *M. caprae*/*M. bovis* ecotypes with no intrinsic pyrazinamide (PZA) resistance, in contrast to the common *M. bovis* strains, detected in samples from Malawi (Guerra‐Assunção et al., 2015), Germany (Friedrich Loeffler Institute) (defined in this study as ecotype I), and China (Orloski, Robbe‐Austerman, Stuber, Hench, & Schoenbaum, 2018) (defined in this study as ecotype II). These findings resulted in a proposed revision of interpretation of the GenoType MTBC test (Hain Lifescience). In regions where tuberculosis is endemic, neonates were vaccinated with the attenuated *M. bovis* strain BCG. In immunocompromised children, this procedure can cause a disease pattern similar to the one of tuberculosis (Hesseling et al., 2006). In order to evaluate a zoonotic risk of MTBC, it is important to rely on a fast and accurate method capable of identification and differentiation of the species of MTBC leading to improved programs in public health surveillance and enhanced food safety.

Various molecular assays exist to differentiate species within MTBC. Nevertheless, many approaches have several limits. Most of the methods as, for example, the commercial GenoType MTBC test are not validated for use on clinical samples (Costa, Amaro, et al., 2014; Kasai, Ezaki, & Harayama, 2000; Niemann, Harmsen, Rüsch‐Gerdes, & Richter, 2000; Pinsky & Banaei, 2008; Pounder et al., 2010; Reddington et al., 2012). Since *Mycobacteria* require many months to obtain considerable growth in culture, methods based on cultivated isolates require much time to determine the correct MTBC species. Other methods are very laborious (Kamerbeek et al., 1997), require expensive equipment (Jagielski et al., 2014), or rely on multiplex qPCR assays (Costa, Amaro, et al., 2014; Halse, Escuyer, & Musser, 2011; Pinsky & Banaei, 2008; Pounder et al., 2010; Reddington et al., 2012). However, the complex composition of a multiplex qPCR can often lead to PCR inhibition due to intricate organ samples of animal tissues.

High‐resolution melting (HRM) approaches are cheap and rapid assays, which are able to detect single nucleotide polymorphism (SNP) according to altered melting temperatures (*T_m_*) of dissociating PCR amplicons (Vossen, Aten, Roos, & Dunnen, 2009). A fluorescent nucleic acid dye is intercalating with the resulting PCR amplicons, which are dissociating upon increase in temperature and thus resulting in a decrease in fluorescence intensity. The determinate of *T_m_* is based on its nucleotide sequence, length, and level of GC. The user‐friendly single‐plex HRM assay can be completed within roughly 2 hr. Moreover, as a major advantage, HRM assays can be performed using samples directly extracted from clinical tissue. High‐resolution melting assays have been used to discriminate various bacteria species (Esteves et al., 2018; Jeffery, Gasser, Steer, & Noormohammadi, 2007; Robertson et al., 2009; Stephens, Inman‐Bamber, Giffard, & Huygens, 2008; Winchell, Wolff, Tiller, Bowen, & Hoffmaster, 2010) or to analyze antibiotic resistance in *M. tuberculosis* (Anthwal et al., 2017; Chen et al., 2011; Yadav et al., 2012). Moreover, HRM assays have been established for differentiation of nontuberculous mycobacteria (NTM) and confining them from MTBC (Issa et al., 2014; Khosravi, Hashemzadeh, Hashemi Shahraki, & Teimoori, 2017; Perng et al., 2012). Some studies combined HRM with multiplex qPCR assays targeting at the region of difference (RD) (Pinsky & Banaei, 2008; Pounder et al., 2010).

We have previously reported the design and evaluation of a HRM assay (HRM assay 1) for the identification and differentiation of MTBC into three groups most relevant for veterinarians (Landolt, Stephan, & Scherrer, 2019). In the present study, two additional HRM assays (HRM assays 2 and 3) were developed with the aim to discern the main members of MTBC. By combining HRM assays 1 and 2 targeting six SNPs on the *gyrB* gene, a two‐step paradigm was obtained, distinguishing *M. tuberculosis*/*M. canettii*, *M. canettii* (rare subtype), *M. africanum*/*M. orygis*/*M. pinnipedii*/*Clade A1*, *M. bovis*/*M. bovis* BCG, *M. caprae*/rare *M. caprae*/*M. bovis* ecotypes, and *M. microti* in cultured isolates and clinical samples. If requested, it is possible to further differentiate *M. bovis*, *M. bovis* BCG, and one of the two rare *M. caprae*/*M. bovis* ecotypes (ecotype I) by conducting another HRM assay (HRM assay 3) based on two SNPs within *gyrA* and thus completing the three‐step paradigm. For most diagnostic applications, however, a combination of HRM assays 1 and 2 will be sufficient.

## 2 MATERIALS AND METHODS

### 2.1 Samples and reference strains

Sixty‐one samples positive for MTBC were collected from 39 different animals (Table 1, Table A1). One wild boar isolate was put at our disposal by courtesy of Lucía de Juan Ferré and Beatriz Romero Martínez. In total, 62 samples including 39 cultured isolates and 23 directly extracted clinical samples were tested with HRM assay 2. High‐resolution melting assay 3 was validated to distinguish between *M. bovis* and *M. bovis* BCG using 15 *M. bovis* isolates and 7 *M. bovis* clinical samples. Positive control samples (*M. microti* ATCC 19422, *M. bovis* BCG Pasteur ATCC 35734, *M. bovis* BCG Tice ATCC 27289, and *M. tuberculosis* H37Rv) were used in each qPCR run. Clinical samples were received in the Laboratory of Veterinary Bacteriology, University of Zurich, between 2013 and 2016.

**Table 1:** MTBC‐positive samples used for the development of the HRM assays 2 and 3

Species	Host	HRM assay 2	HRM assay 3
		No. of isolates	No. of isolates
Cultured material (*n* = 39)			
*M. tuberculosis*	Elephant	3	
*M. caprae*	Cow	7	
*M. bovis*	Cow	15	15
*M. microti*	Cat	8	
*M. microti*	Alpaca	3	
*M. microti*	Llama	2	
*M. microti*	Wild boar (Spain)	1	
Clinical samples (*n* = 23)			
*M. tuberculosis*	Elephant	2	
*M. caprae*	Cow	4	
*M. bovis*	Cow	7	7
*M. microti*	Cat	5	
*M. microti*	Alpaca	3	
*M. microti*	Llama	2	
Total		62	22

Note

Thirty‐nine isolates obtained from cultured material, whereas 23 samples were clinical samples directly extracted from tissue samples. Sixty‐one samples were derived from Switzerland, whereas one isolate originated from Spain.

### 2.2 Culture and DNA extraction

Sample preparation, culture, and DNA extraction were conducted as described in a former study (Ghielmetti et al., 2017). GenoType MTBC test (Hain Lifescience), spoligotyping (Ruettger et al., 2012), and multilocus variable number tandem repeat analysis using an internationally established 24‐loci panel (Supply et al., 2006) were used for species identification of cultured isolates. Standard biosecurity procedures were followed for handling of samples.

### 2.3 HRM development

**Figure 1:**
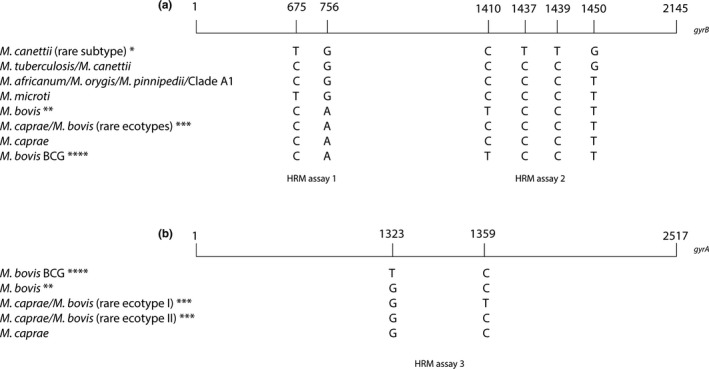
Differentiation of MTBC based on eight different single nucleotide polymorphisms (SNPs). Numbers represent the position of the SNP in relation to the start codon of *gyrB* and *gyrA*, respectively. (a) Six SNPs on *gyrB* are represented. HRM assay 1 (Landolt et al., 2019) allows the distinction between *M. tuberculosis*/*M. canettii*/*M. africanum*/*M. orygis*/*M. pinnipedii*/Clade A1, *M. microti*/*M. canettii* (rare subtype) and *M. bovis*/*M. bovis* BCG/*M. caprae*/rare *M. caprae*/*M. bovis* ecotypes. HRM assay 2 can differentiate between *M. tuberculosis*/*M. canettii*, *M. canettii* (rare subtype), *M. africanum*/*M. orygis*/*M. pinnipedii*/Clade A1/*M. microti*/*M. caprae*/rare *M. caprae*/*M. bovis* ecotypes, and *M. bovis*/*M. bovis* BCG. Combining the results of HRM assays 1 and 2, six groups can be distinguished: *M. canettii* (rare subtype), *M. tuberculosis*/*M. canettii*,* M. africanum*/*M. orygis*/*M. pinnipedii*/Clade A1,* M. microti*,* M. caprae*/rare *M. caprae*/*M. bovis* ecotypes, and *M. bovis*/*M. bovis* BCG. (b) Two SNPs on positions 1,323 and 1,359 of *gyrA* are illustrated indicating the differentiation between *M. bovis*, *M. bovis* BCG, and rare *M. caprae*/*M. bovis* ecotype I. *Rare subtype, highly recombinogenic strain, intrinsic pyrazinamide (PZA) resistance. **Intrinsic PZA resistance. ***No intrinsic PZA resistance. ****Intrinsic PZA and cycloserine resistance

High‐resolution melting development of assay 1 was described in a former study (Landolt et al., 2019). The differentiation of the main members of MTBC is performed applying a paradigm of a three‐reaction HRM assay based on four SNPs located on *gyrB* (base pair positions 675, 756, 1,410, and 1,450 [Niemann et al., 2000] and one SNP on *gyrA* [base pair position 1,323]). In order to extend the obtained SNP differentiation scheme with the recently described two rare ecotypes of *M. caprae*/*M. bovis* (ecotypes I and II), a rare *M. canettii* subtype (Loiseau et al., 2019) and the animal‐adapted MTBC clades (Brites et al., 2018), two additional SNPs on *gyrB* (base pair positions 1,437 and 1,439), and one SNP on *gyrA* (base pair position 1,359) were included in the assay design (Figure 1).

### 2.4 Primer of HRM assays

Primers were designed based on alignments of the available sequences of *gyrB* and *gyrA* from MTBC (GenBank accession numbers: *gyrB M. bovis* AB014184.1, *gyrB M. microti* AB014205.1, *gyrB M. tuberculosis* X78888.1, *gyrB M. africanum* FR878060.1, *gyrB M. caprae* CP016401.1, *gyrA M. bovis* LT708304.1, and *gyrA* *M. bovis* BCG Pasteur AM408590.1). The designed primers amplify a conserved 107‐base pair (bp) fragment within *gyrB* comprising two SNPs and *gyrA* comprising one SNP, respectively (Figure 1). Gene specificity of all primers was confirmed by NCBI BLAST searches. Primers were HPLC‐purified and synthesized by Microsynth. Three primer pairs were designed for the three‐reaction HRM paradigm (Table 2).

**Table 2:** Primers used for the three‐reaction HRM paradigm

Assay	Primer	Target	Amplicon	Primer sequence (5′ → 3′)	Reference
HRM assay 1	HRM_gyrB_for HRM_gyrB_rev	*gyrB*	144 bp	CGGCTCGAAGTCGAGATCAAG TTCGAAAACAGCGGGGTCG	Landolt et al. (2019)
HRM assay 2	HRM_gyrB2_for HRM_gyrB2_rev	*gyrB*	107 bp	CAAATCGTTTGTGCAGAAGGTCTG CTTGCGCCGAGGACACAG	This study
HRM assay 3	HRM_gyrA_for HRM_gyrA_rev	*gyrA*	107 bp	AGGCAATCCTGGACATGCAG GATGTCTTCCAGATCGGCGATC	This study

### 2.5 qPCR and melting conditions

Each HRM assay was processed separately on a Rotor‐Gene Q system (Qiagen) with the Type‐it HRM PCR Kit (Qiagen). The qPCRs were performed as described previously (Landolt et al., 2019). High‐resolution melting ramping from 76°C to 93°C was applied. Fluorescence data were measured every 2 s at 0.1°C increments generating specific melting curves. Reference strains *M. microtii* ATCC 19422, *M. bovis* BCG Pasteur ATCC 35734, and *M. tuberculosis* H37Rv were used as melting curve standards and positive controls. Additionally, for HRM assay 3, *M. bovis* BCG Tice ATCC 27289 was used. As a negative control, ultrapure water was included in each experiment. Rotor‐Gene Q Software 2.3.1 (Qiagen) was used for data analysis to generate normalized and difference plots as described in a previous study (Landolt et al., 2019). To prevent false‐negative results possibly deriving from inhibition, clinical samples were analyzed in duplicate undiluted and in the form of a 1:5 dilution. The cultured isolates were analyzed at concentrations between 100 pg and 10 ng.

To investigate the intra‐ and interassay variability of the *T_m_*, illustrating the repeatability of the developed HRM assays, a randomly chosen subset of 22 cultured isolates and 18 clinical specimens for HRM assays 2 and 9 cultured isolates and 7 clinical specimens for HRM assay 3 were tested, respectively. The variability assays were conducted in triplicates in three single runs at three different days.

### 2.6 Specificity

To check for possible nonspecific signals of HRM assays 2 and 3, 41 different NTM, *Nocardia paucivorans*, *Escherichia coli*, and *Streptococcus suis* were tested (Table A2).

### 2.7 Sensitivity

The analytical sensitivity of HRM assay 2 was measured by triplicate testing of a 10‐fold dilution series of DNA isolated from strains *M. microti* ATCC 19422, *M. bovis* BCG Pasteur ATCC 35734, *M. caprae* clinical isolate ZH22914, and *M. tuberculosis* H37Rv with known concentrations in genome equivalents (GE). The analytical sensitivity of HRM assay 3 was tested for *M. bovis* BCG Pasteur ATCC 35734 and the clinical isolate *M. bovis* ZH20665 in an analogous manner by analyzing 10‐fold DNA dilution series. Based on an estimated genome size of 4.4 Mb for members of MTBC, 1 GE correlates with a DNA quantity of 4.8 fg. The slope of the resulting standard curve corresponded to the amplification efficiency of each tested strain. The limit of detection (LOD) was defined as lowest dilution for samples with successful PCR amplification of all triplicates having a Ct < 38 and a standard deviation of ≤ 0.5.

## 3 RESULTS

### 3.1 HRM of cultured isolates

**Figure 2:**
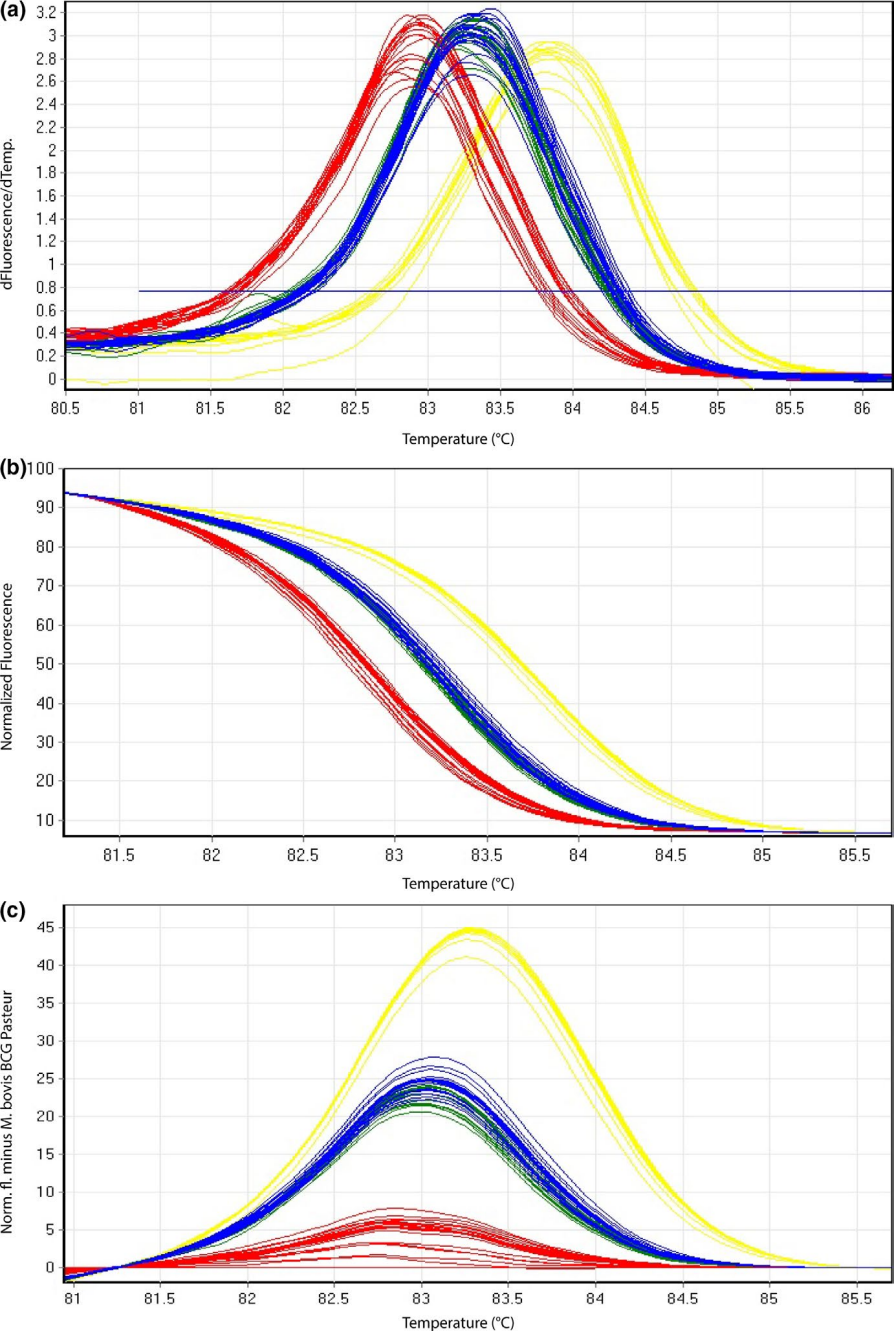
Representative high‐resolution melting graphs corresponding to one high‐resolution melting analysis of a subset of cultured samples (*n* = 22) of HRM assay 2. Curves of tested samples previously identified as *M. tuberculosis* are shown in yellow,* M. microti* in blue, *M. bovis*/*M. bovis* BCG in red, and *M. caprae* in green. (a) Melting curves; (b) normalized plot; and (c) difference plot in relation to *M. bovis* BCG Pasteur ATCC 35734

**Figure 3:**
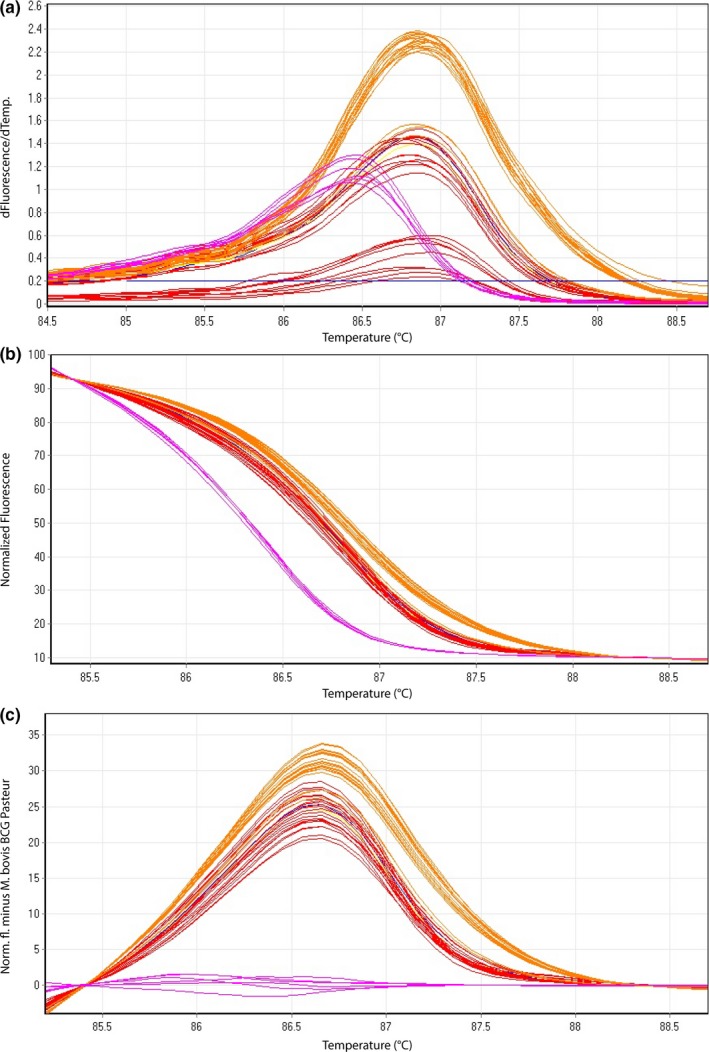
Representative high‐resolution melting graphs corresponding to one high‐resolution melting analysis of a subset of cultured samples (*n* = 9) and clinical specimens (*n* = 7) of HRM assay 3. Curves of tested samples previously identified as *M. bovis* BCG are shown in pink, cultured samples of *M. bovis* in red, and clinical specimen of *M. bovis* in orange. (a) Melting curves; (b) normalized plot; and (c) difference plot in relation to *M. bovis* BCG Pasteur ATCC 35734

All cultured isolates tested were amplified successfully yielding a melting curve. The resulting species‐specific *T_m_* (Tables 3 and 4, Tables A3 and A4) of corresponding melting curves (Figures 2 and 3) from sample subsets used for the determination of the intra‐ and interassay variability clearly represent three independent groups in the case of HRM assay 2 and two distinct groups with HRM assay 3, respectively. Obtained *T_m_* ranges between the different groups are very close to each other impeding a clear differentiation between members of MTBC solely based on *T_m_*. In contrast, the illustration of dissociating curves in the form of difference plots (Figures 2c and 3c) as well as normalized plots (Figures 2b and 3b) allowed a clear differentiation between MTBC species.

**Table 3:** Intra‐ and interassay variability of HRM assay 2 of cultured samples

	Run 1		Run 2		Run 3		Interassay	
	*T_m_*	CV%	*T_m_*	CV%	*T_m_*	CV%	*T_m_*	CV%
*M. tuberculosis* H37Rv	83.55		83.75		83.6		83.63	0.12
*M. bovis* BCG Pasteur ATCC 35734	82.62		82.77		82.70		82.7	0.09
*M. microti* ATCC 19422	83.13		83.25		83.28		83.22	0.10
*M. tuberculosis* (*n* = 3)	83.56 ± 0.04	0.01	83.84 ± 0.04	0.02	83.66 ± 0.06	0.04	83.70 ± 0.18	0.17
*M. bovis* (*n* = 6)	82.70 ± 0.10	0.04	82.93 ± 0.08	0.03	82.74 ± 0.06	0.02	82.80 ± 0.20	0.14
*M. caprae* (*n* = 6)	83.12 ± 0.09	0.03	83.27 ± 0.09	0.02	83.23 ± 0.06	0.02	83.19 ± 0.16	0.11
*M. microti* (*n* = 7)	83.10 ± 0.08	0.02	83.34 ± 0.09	0.04	83.29 ± 0.04	0.01	83.23 ± 0.21	0.14

Note

Mean values and standard deviation of melting temperatures (*T_m_*) of a randomly chosen subset of cultured samples are listed. Corresponding coefficients of variation (CV) in % are indicated for each MTBC species tested.

**Table 4:** Intra‐ and interassay variability of HRM assay 3 of cultured samples

	Run 1		Run 2		Run 3		Interassay	
	*T_m_*	CV%	*T_m_*	CV%	*T_m_*	CV%	*T_m_*	CV%
*M. tuberculosis* H37Rv	86.82		86.82		86.85		86.83	0.02
*M. microti* ATCC 19422	86.83		86.83		86.83		86.83	0
*M. bovis* BCG Pasteur ATCC 35734	86.42		86.38		86.42		86.41	0.03
*M. bovis* (*n* = 7)	86.83 ± 0.50	0.01	86.83 ± 0.08	0.02	86.83 ± 0.10	0.03	86.83 ± 0.10	0.03
*M. bovis* BCG (*n* = 2)	86.39 ± 0.06	0.03	86.41 ± 0.03	0.03	86.45 ± 0.03	0.03	86.41 ± 0.08	0.04

Note

Mean values and standard deviation of melting temperatures (*T_m_*) of a randomly chosen subset of cultured samples are listed. Corresponding coefficients of variation (CV) in % are indicated for each MTBC species tested.

High‐resolution melting assay 2 differentiates three groups, namely *M. tuberculosis*, *M. microti*/*M. caprae*, and *M. bovis*/*M. bovis* BCG. The obtained intra‐assay coefficients of variation (CVs) and the interassay CVs were ranging between 0.01%–0.04% and 0.11%–0.17%, respectively (Table 3, Table A3), demonstrating highly reproducible and robust assays. Species identification results of all 39 (100%) tested cultured isolates were in accordance with the species classification of GenoType MTBC test (Hain Lifescience) results.

High‐resolution melting assay 3 differentiates *M. bovis* from *M. bovis* BCG. The intra‐assay CVs and the interassay CVs yielded *T_m_* values ranging between 0.01%–0.03% and 0.03%–0.04%, respectively (Table 4, Table A4). Species identification results of all 15 (100%) tested cultured isolates were in agreement with the GenoType MTBC test (Hain Lifescience) results.

### 3.2 HRM of clinical samples

**Figure 4:**
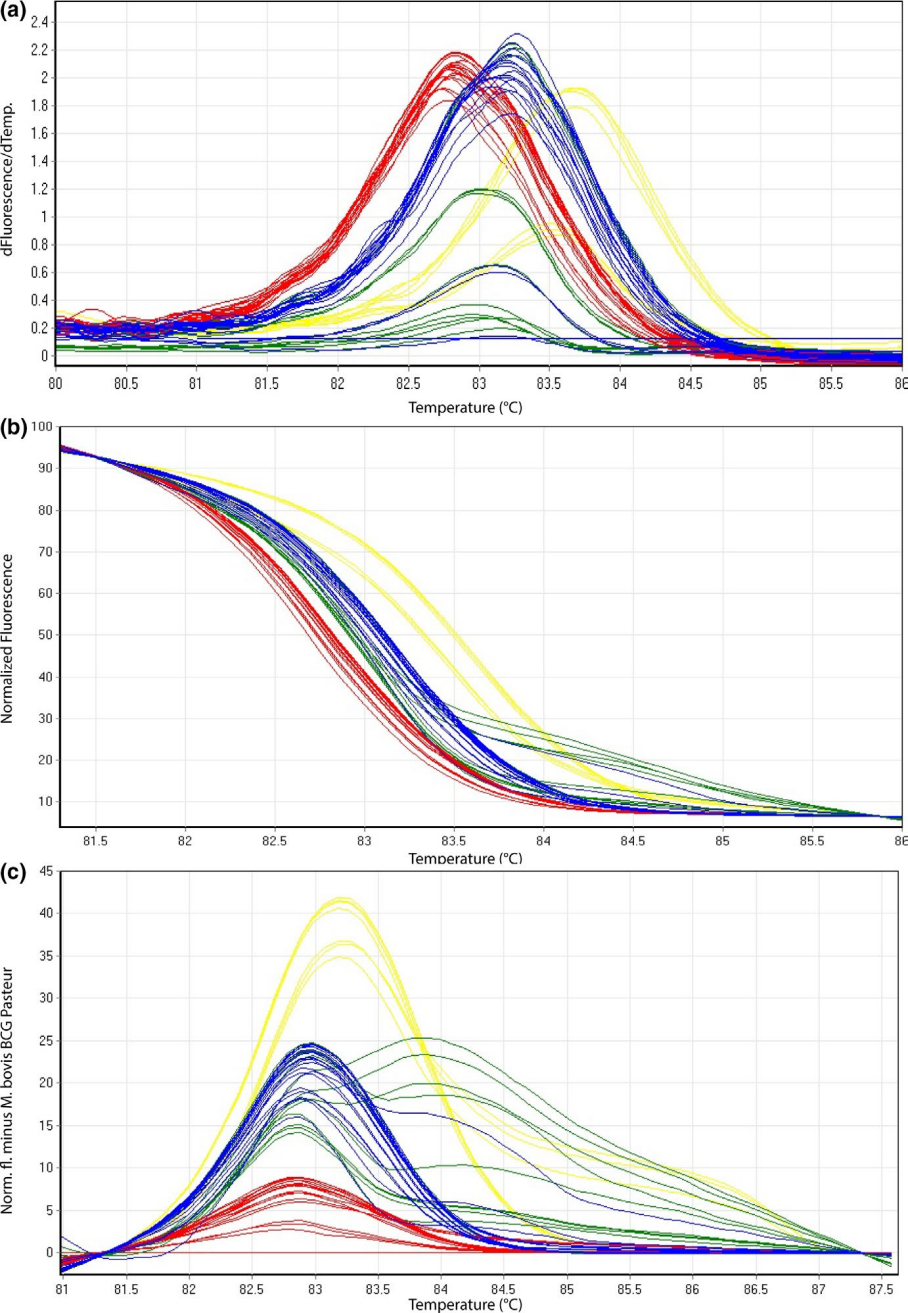
Representative high‐resolution melting graphs corresponding to one high‐resolution melting analysis of a subset of clinical specimens (*n* = 18) for HRM assay 2. Curves of tested samples previously identified as *M. tuberculosis* are shown in yellow,* M. microti* in blue, *M. bovis*/*M. bovis* BCG in red, and *M. caprae* in green. (a) Melting curves; (b) normalized plot; and (c) difference plot in relation to *M. bovis* BCG Pasteur ATCC 35734

For 22/23 (96%) clinical samples tested in HRM assay 2, obtained normalized and difference plots (Figure 4b,c) showed the appearance of three distinct groups, namely *M. tuberculosis*, *M. microti*/*M. caprae*, and *M. bovis*/*M. bovis* BCG in accordance with the results of GenoType MTBC test (Hain Lifescience). One sample revealed significantly lower *T_m_* values compared with the other results. This clinical sample (samples 17–1,063), however, has not yet been successfully cultured and is in the progress of further investigations. High‐resolution melting assay 3 showed a clear distinction of all cultured isolates as well as directly isolated clinical samples of *M. bovis* from *M. bovis* BCG in 100% concordance with the GenoType MTBC test (Hain Lifescience) results.

In HRM assay 2, intra‐ and interassay CVs were between 0.02%–0.04% and between 0.12%–0.19%, respectively (Table 5, Table A5).

**Table 5:** Intra‐ and interassay variability of HRM assay 2 of clinical specimens

	Run 1		Run 2		Run 3		Interassay	
	*T_m_*	CV%	*T_m_*	CV%	*T_m_*	CV%	*T_m_*	CV%
*M. tuberculosis* H37Rv	83.85		83.67		83.58		83.70	0.16
*M. bovis* BCG Pasteur ATCC 35734	82.82		82.73		82.62		82.72	0.12
*M. microti* ATCC 19422	83.30		83.25		83.08		83.21	0.14
*M. tuberculosis* (*n* = 2)	83.71 ± 0.13	0.03	83.60 ± 0.10	0.03	83.52 ± 0.09	0.03	83.63 ± 0.20	0.12
*M. bovis* (*n* = 6)	82.97 ± 0.10	0.04	82.82 ± 0.07	0.02	82.70 ± 0.10	0.03	82.84 ± 0.24	0.19
*M. caprae* (*n* = 4)	83.24 ± 0.14	0.02	83.11 ± 0.16	0.02	82.97 ± 0.12	0.03	83.11 ± 0.26	0.15
*M. microti (n* = 6)	83.29 ± 0.09	0.02	83.19 ± 0.09	0.03	83.00 ± 0.10	0.04	83.14 ± 0.24	0.16

Note

Mean values and standard deviation of melting temperatures (*T_m_*) of a randomly chosen subset of clinical specimen are listed. Corresponding coefficients of variation (CV) in % are indicated for each MTBC species tested.

High‐resolution melting assay 3 showed intra‐assay CVs between 0.01% and 0.02% and an interassay CV of 0.04% (Table 6, Table A4).

**Table 6:** Intra‐ and interassay variability of HRM assay 3 of clinical specimens

	Run 1		Run 2		Run 3		Interassay	
	*T_m_*	CV%	*T_m_*	CV%	*T_m_*	CV%	*T_m_*	CV%
*M. tuberculosis* H37Rv	86.82		86.82		86.85		86.83	0.02
*M. microti* ATCC 19422	86.83		86.83		86.83		86.83	0
*M. bovis* BCG Pasteur ATCC 35734	86.45		86.42		86.47		86.41	0.03
*M. bovis* (*n* = 7)	86.86 ± 0.04	0.01	86.84 ± 0.07	0.02	86.88 ± 0.08	0.02	86.86 ± 0.09	0.04

Note

Mean values and standard deviation of melting temperatures (*T_m_*) of a randomly chosen subset of clinical specimen are listed. Corresponding coefficients of variation (CV) in % are indicated for each MTBC species tested.

### 3.3 Specificity

Forty‐one NTM and *N. paucivorans*, *E. coli*, and *S. suis* were tested for specificity of HRM assay 2 resulting in no melting curves or melting curves with entirely different *T_m_* in respect to *T_m_* deriving from samples of MTBC. In HRM assay 3, some NTM showed similar curves and *T_m_* values. Therefore, HRM assay 3 is recommended to be applied only after identification of *M. bovis* or *M. bovis* BCG.

### 3.4 Sensitivity

**Figure A1:**
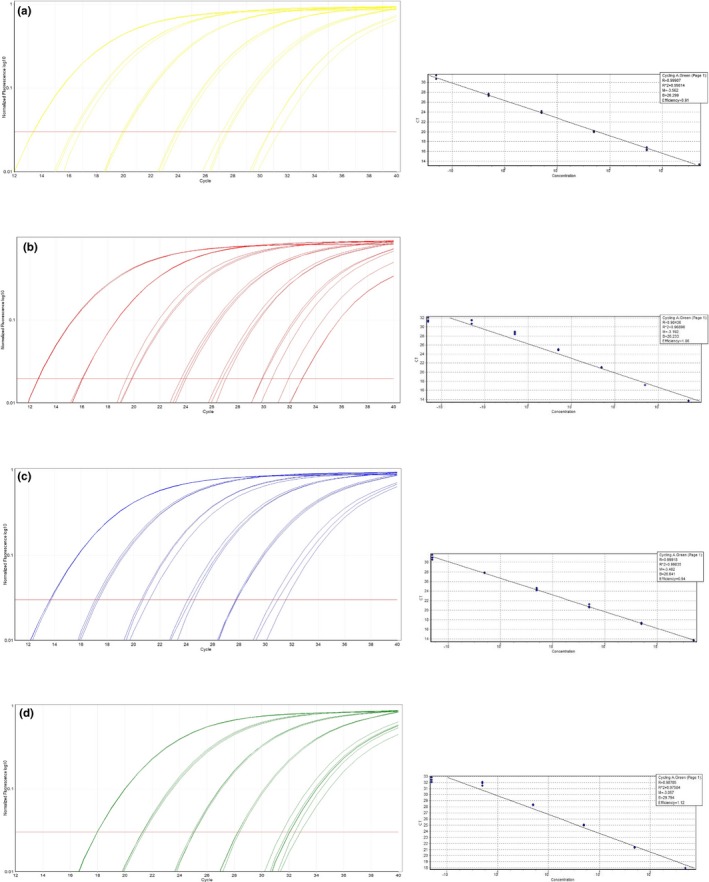
qPCR amplification curves and standard curves of the serial dilution using (a) *M. tuberculosis* H37Rv; (b) *M. bovis* BCG Pasteur ATCC 35734; (c) *M. microti* ATCC 19422; and (d) *M. caprae* ZH 22914 for high‐resolution melting (HRM) assay 2

**Figure A2:**
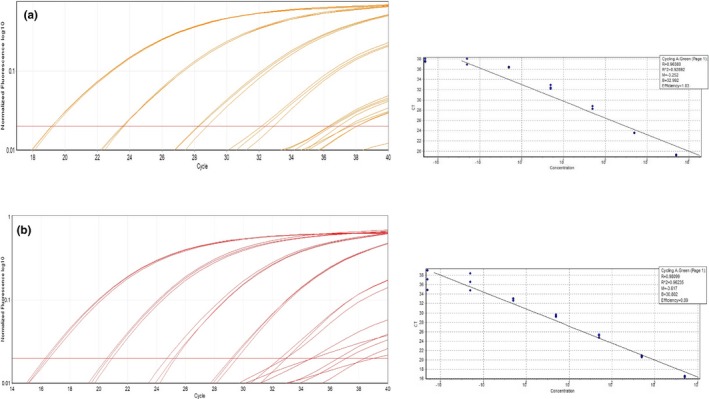
qPCR amplification curves and standard curves of the serial dilution using (a) *M. bovis* BCG Pasteur ATCC 35734 and (b) *M. bovis* for high‐resolution melting (HRM) assay 3

For HRM assay 2, efficiencies of the qPCR were 94% for *M. microti*, 97% for *M. bovis*, 112% for *M. caprae*, and 91% for H37Rv (Figure A1). In HRM assay 3, efficiencies were determined to be 89% for *M. bovis* BCG and 103% for *M. bovis* (Figure A2).

High‐resolution melting assay 2 showed a LOD for the lowest dilution of which the acceptance criteria (standard deviation < 0.5 and Ct value < 38) were complied with 10 GE, corresponding to 50 fg of template DNA in the qPCR, for *M. tuberculosis*,* M. caprae*,* M. microti*, and *M. bovis* (Table 7). The LOD of HRM assay 3 was 100 GE for both *M. bovis* and *M. bovis* BCG (Table 8).

**Table 7:** Limit of detection (LOD) of the qPCR of HRM assay 2

MTBC member	Genome equivalents	Ct	*SD*
*M. tuberculosis* H37Rv	1,000,000	13.37	0.01
	100,000	16.49	0.28
	10,000	19.99	0.08
	1,000	23.96	0.17
	100	27.46	0.22
	**10**	**30.92**	**0.40**
	1	33.06	1.15
*M. bovis* BCG pasteur ATCC 35734	1,000,000	12.62	0.02
	100,000	16.02	0.03
	10,000	19.69	0.24
	1,000	23.82	0.19
	100	26.83	0.24
	**10**	**30.11**	**0.43**
	1	32.6	0.61
*M. microti* ATCC 19422	1,000,000	13.68	0.05
	100,000	17.20	0.13
	10,000	20.80	0.33
	1,000	24.34	0.25
	100	27.77	0.04
	**10**	**31.01**	**0.49**
	1	33.19	0.52
*M. caprae* ZH 22914	1,000,000	17.96	0.01
	100,000	21.31	0.07
	10,000	25.02	0.06
	1,000	28.28	0.07
	100	31.79	0.29
	**10**	**32.42**	**0.38**
	1	34.09	0.98

Note

Determination of Ct values and its standard deviation (*SD*) of 3 replicates for a dilution series ranging from 1 to 1,000,000 genome equivalents using reference strains *M. tuberculosis* H37Rv, *M. bovis* Pasteur ATCC 35734, *M. microti* ATCC 19422, and the clinical specimen *M. caprae* ZH 22914. Bold represents the determined LOD for the lowest dilution of which the acceptance criteria (standard deviation < 0.5 and Ct value < 38) were fulfilled.

**Table 8:** Limit of detection (LOD) of the qPCR of HRM assay 3

MTBC member	Genome equivalents	Ct	*SD*
*M. bovis* ZH 20655	1,000,000	19.19	0.07
	100,000	23.57	0.04
	10,000	28.41	0.31
	1,000	32.45	0.39
	**100**	**36.37**	**0.10**
	10	37.46	0.80
	1	37.67	0.28
*M. bovis* BCG pasteur ATCC 35734	1,000,000	16.41	0.14
	100,000	20.72	0.18
	10,000	25.06	0.31
	1,000	29.34	0.20
	**100**	**32.83**	**0.24**
	10	35.57	1.84
	1	37.00	2.10

Note

Determination of Ct values and its standard deviation (*SD*) of 3 replicates for a dilution series ranging from 1 to 1,000,000 genome equivalents using reference strains *M. bovis* BCG Pasteur ATCC 35734 and the clinical specimen *M. bovis* ZH 20665. Bold represents the determined LOD for the lowest dilution of which the acceptance criteria (standard deviation < 0.5 and Ct value < 38) were fulfilled.

## 4 DISCUSSION

In the present study, the establishment of a three‐reaction HRM paradigm in the form of three HRM assays is described, which can rapidly differentiate the main species of MTBC. *M. microti*,* M. tuberculosis*,* M. caprae*,* M. bovis*, and *M. bovis* BCG were demonstrated to undoubtedly and consistently be differentiated from each other by distinctive difference plots (Figures 2c, 3c, and 4c). Based on the recently published suggestion of revising the Hain GenoType MTBC test interpretation, proposing eight possible binding patterns including novel ecotypes/subtypes (Loiseau et al., 2019), the three‐reaction HRM paradigm can potentially reveal the same eight different groups. However, the Hain test is only validated for cultured samples and remains a very costly and time‐consuming approach comprising 13 different probes.

The clear advantage of the developed three‐reaction HRM paradigm approach compared with previous studies (Costa, Amaro, et al., 2014; Costa, Botelho, Couto, Viveiros, & Inácio, 2014; Halse et al., 2011; Pinsky & Banaei, 2008; Pounder et al., 2010; Reddington et al., 2012) is the achievement of an inexpensive, rapid as well as easy to use single‐plex method, which can be used for cultured samples as well as for clinical samples. In approximately 2 hr, a sample can be identified as member of MTBC and assigned to the correct species, which is a benefit comparing to methods, which are based on time‐consuming procedures (Kamerbeek et al., 1997) or require cultured samples (Kasai et al., 2000; Niemann et al., 2000). An additional advantage of this three‐reaction HRM paradigm aiming at different loci lays in the fact that it is an adaptive approach, which opens the possibility for individual combinations of primer pairs depending on the question raised. In case of a probable detection of *M. orygis* strains and therefore a desired differentiation from *M. africanum*, there is potential to design a further primer pair, which would allow detecting a mutation at codon 329 of *gyrB* (Huard et al., 2006) representing a unique SNP in *M. orygis*.

The only drawback of the described HRM approach compared to the Hain test is the inability to differentiate three strains originating from China (Orloski et al., 2018) (*M. caprae*/*M. bovis* ecotype II) from *M. caprae*. To resolve this problem, it would be possible to expand the assay by designing a novel primer pair covering a SNP at base pair position 1,310 of *gyrB* and thus reliably identifying *M. caprae*/*M. bovis* ecotype II. Since *M. caprae*/*M. bovis* ecotype II is a rare type of strains, it can be neglected in routine diagnostic laboratories, where it is essential to rely on simply performable assays. Moreover, an additional differentiation of *M. caprae*/*M. bovis* ecotype II from *M. caprae* has no advantage in respect to the choice of antibiotic treatment, because both strain types are not intrinsically resistant to PZA.

**Figure 5:**
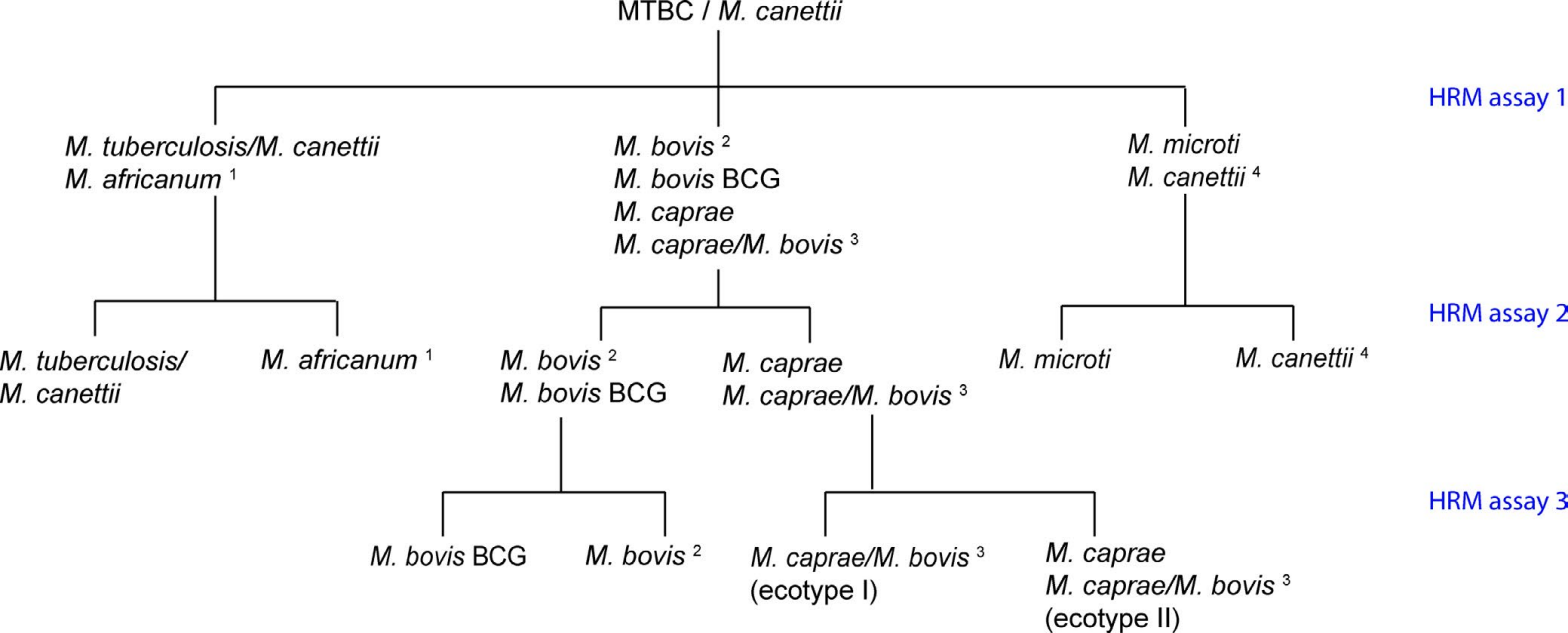
HRM assay 1 (Landolt et al., 2019) allows the distinction between *M. tuberculosis*/*M. canettii*/*M. africanum*/*M. orygis*/*M. pinnipedii*/Clade A1, *M. microti*/*M. canettii* (rare subtype), and *M. bovis*/*M. bovis* BCG/*M. caprae*/rare *M. caprae*/*M. bovis* ecotypes. A combination of HRM assays 1 and 2 is leading to six groups (*M. tuberculosis*/*M. canettii*,* M. africanum*/*M. orygis*/*M. pinnipedii*/Clade A1,* M. microti*,* M. canettii* (rare subtype),* M. caprae*/rare *M. caprae*/*M. bovis* ecotypes I and II, and *M. bovis*/*M. bovis* BCG). By performing HRM assay 3, *M. bovis*, *M. bovis* BCG, and rare *M. caprae*/*M. bovis* ecotype I can further be distinguished. 1. *M. africanum* not distinguishable in *gyrA* and *gyrB* from *M. orygis*,* M. pinnipedii*, Clade A1 (Dassie bacillus,* M. mungi*, Chimpanzee bacillus, *M. suricattae*) (Brites et al., 2018). 2. Frequent subtype, intrinsic pyrazinamide (PZA) resistance. 3. Rare ecotypes, no intrinsic PZA resistance. 4. Rare subtype, highly recombinogenic

By a stepwise combination of three independent HRM assays, it is possible to differentiate the species of MTBC firstly into three groups (*M. microti*/*M. canettii* (rare subtype),* M. tuberculosis*/*M. africanum*/*M. orygis*/*M. pinnipedii*/Clade A1, and *M. caprae*/*M. bovis*/*M. bovis* BCG/rare *M. caprae*/*M. bovis* ecotypes), secondly into six groups (*M. microti*,* M. tuberculosis*/*M. canettii*,* M. canettii* (rare subtype), *M. africanum*/*M. orygis*/*M. pinnipedi*/Clade A1, *M. caprae*/rare *M. caprae*/*M. bovis* ecotypes,* and M. bovis*/*M. bovis* BCG), and finally on *gyrA*,* M. bovis* BCG, *M. bovis*, and rare *M. caprae*/*M. bovis* ecotype I can further be separated leading to a clear differentiation into the main human‐ and veterinary‐associated MTBC species (Figure 5). The three HRM assays can be performed either consecutively or in parallel since the qPCR conditions are equal. The interpretation of the three‐reaction paradigm is straightforward and simple to achieve. For routine laboratories, a simple combination of HRM assays 1 and 2 will lead to a rapid detection and differentiation of the most significant agents of tuberculosis appearing worldwide.

*T_m_* ranges deriving from HRM assays are partially overlapping (Tables 3, 4, 5, 6). Therefore, based solely on *T_m_* it is not possible to clearly differentiate the members of MTBC. However, by transforming melting curves into normalized and difference plots using algorithms of the Rotor‐Gene Q Software 2.3.1 (Qiagen), the members of MTBC can be separated into distinct groups (Figures 2, 3, 4). Thereby, the species‐specific melting profiles showed an explicit behavior. The presented HRM assays identified MTBC‐positive cultured isolates in accordance with the results of the GenoType MTBC test.

The clinical samples were in agreement of 96% with respect to the GenoType MTBC test. One sample (4%) deriving from an alpaca, however, showed a nonspecific melting curve preventing a correct assessment of the sample to MTBC applying the developed HRM assays. A possible explanation of this interference of the melting curve could be correlated to the inhibitory substances of the complex sample mixture originating from different tissues including lymph node, lung, heart, liver, and cervical vertebra. Investigations to unravel this finding were not successful yet. Although all remaining 22 clinical samples deriving from lymph nodes, lung, or liver tissues showed an unambiguous and correct result (Table A1), it is important to mention that samples containing very little amount of target DNA material (as obtained in two tested *M. caprae* samples and one *M. microti* clinical sample) display a weak fluorescence signal (Figure 4a) and therefore may lead to irregular shapes of the normalized—(Figure 4b) and difference—(Figure 4c) plots in comparison with all other samples enclosing high amounts of MTBC DNA. Such irregular patterns of normalized and difference plots visualize the detection limit of a HRM assay when testing directly extracted clinical samples.

Summarizing the obtained data, both HRM assays 2 and 3 showed a very good reproducibility with small variations of *T_m_*, which is demonstrated in very low values of intra‐ and interassay CVs. Since HRM assays 1 and 2 are proven to be 100% specific, they can be used unambiguously for the identification and differentiation of MTBC. Moreover, the assays yielded PCR efficiencies of more than 91% and 89%, respectively. The sensitivity of HRM assay 2 showing a LOD of 10 GE is adequate. The LOD of HRM assay 3 is slightly higher with a measured LOD of 100 GE. However, since the tested collective of samples did not cover species of all eight genotype groups, it is suggested to further evaluate the assay by testing a more extensive collection of isolates.

## 5 CONCLUSION

The developed three‐reaction HRM paradigm is a quick, sensitive, and specific assay for differentiation of MTBC between the main species highly relevant in human and veterinary diagnostics namely *M. tuberculosis*/*M. canettii*,* M. canettii* (rare subtype), *M. africanum*/*M. orygis*/*M. pinnipedii*/*Clade A1*, *M. microti*,* M. caprae*/rare *M. caprae*/*M. bovis* ecotype II,* M. bovis*, *M. bovis* BCG, and rare *M. caprae*/*M. bovis* ecotype I extracted from clinical samples and from isolates. Several months of cultivation time may be saved by using these potent HRM assays. Since most species within MTBC are implicated in human infections (Huard et al., 2006), it is of advantage to have early knowledge of transmission of tuberculosis for consequently choosing an appropriate drug therapy for humans or a proper eradication strategy when dealing with veterinary samples. Tuberculosis surveillance policies and public health management depend on powerful and affordable diagnostic tools such as this paradigm of a three‐reaction HRM assay, which could be easily implemented in laboratories worldwide.

## CONFLICT OF INTEREST

None declared.

## AUTHOR CONTRIBUTIONS

PL and SS conceptualized, drafted, and investigated the data; involved in formal analysis; and wrote the original manuscript. MJAS edited and provided sequencing data. RS and SS wrote, reviewed, and edited the manuscript.

## ETHICS STATEMENT

The recommendations of Swiss federal regulations (TSV 916.401 and VSFK 817.190) were followed. The animal samples were analyzed in the context of a monitoring program of lymph nodes aiming at an early recognition of bovine tuberculosis and NTM infections. No animals were killed for the purposes of this research project, and no ethical approval was required.

## Data Availability

Raw data sets from intra‐ and interassay variability runs are comprehended in the appendix. On request, additional raw data can be obtained from the corresponding author.

## APPENDIX A

**Table A1:** MTBC‐positive isolates used for the development of the high‐resolution melting (HRM) assays 2 and 3

Sample	Species	Origin	Specimen	Culture?	Clinical specimen?
*M. bovis* (15)					
20175	*M. bovis*	Cow	Lung	Cultured	Not available
20482	*M. bovis*	Cow	Lung, lymph node pool	Cultured	Not available
20531	*M. bovis*	Cow	Lymph node pool	Cultured	Not available
20593	*M. bovis*	Cow	Lymph node pool	Cultured	Clinical specimen
20594	*M. bovis*	Cow	Lymph node pool	Cultured	Clinical specimen
20596	*M. bovis*	Cow	Lymph node pool	Cultured	Clinical specimen
20597	*M. bovis*	Cow	Lymph node pool	Cultured	Not available
20599	*M. bovis*	Cow	Lymph node pool	Cultured	Not available
20600	*M. bovis*	Cow	Lymph node pool	Cultured	Clinical specimen
20606	*M. bovis*	Cow	Lymph node pool	Cultured	Clinical specimen
20608	*M. bovis*	Cow	Lymph node pool	Cultured	Clinical specimen
20609	*M. bovis*	Cow	Lymph node pool	Cultured	Clinical specimen
20665	*M. bovis*	Cow	Lymph node pool	Cultured	Not available
22539	*M. bovis*	Cow	Lymph node	Cultured	Not available
22667	*M. bovis*	Cow	Lymph node	Cultured	Not available
*M. caprae* (7)					
14‐13	*M. caprae*	Cow	Lymph node	Cultured	Not available
22914	*M. caprae*	Cow	Lymph node pool	Cultured	Clinical specimen
22971	*M. caprae*	Cow	Lymph node pool	Cultured	Clinical specimen
22848	*M. caprae*	Cow	Liver, lung, lymph node pool	Cultured	Clinical specimen
22966	*M. caprae*	Cow	Lymph node pool	Cultured	Not available
13‐450	*M. caprae*	Cow	Lymph node	Cultured	Not available
13‐162	*M. caprae*	Cow	Lymph node	Cultured	Clinical specimen
*M. microti* (15)					
22928	*M. microti*	Cat	Lung, lymph node pool	Cultured	Clinical specimen
15‐1765	*M. microti*	Cat	Lung	Cultured	Clinical specimen
15‐342	*M. microti*	Alpaca	Lymph node	Cultured	Clinical specimen
14‐58	*M. microti*	Cat	Lung	Cultured	Clinical specimen
14‐690	*M. microti*	Alpaca	Liver	Cultured	Clinical specimen
17‐2287	*M. microti*	Alpaca	Spleen	Cultured	Not available
15817	*M. microti*	Lama	Lymph node	Cultured	Clinical specimen
15‐1955	*M. microti*	Cat	Lymph node	Cultured	Clinical specimen
16‐2156	*M. microti*	Cat	Bronchoalveolar lavage	Cultured	Not available
1522744	*M. microti*	Cat	Lung, lymph node pool	Cultured	Not available
16‐1347	*M. microti*	Cat	Lung	Cultured	Not available
17‐1084	*M. microti*	Cat	Lymph node, skin	Cultured	Clinical specimen
17‐1063	*M. microti*	Alpaca	Lymph node, lung, heart, liver, cervical vertebra	Culture ongoing	Clinical specimen
17‐549	*M. microti*	Lama	Liver	Cultured	Clinical specimen
MI16	*M. microti*	Wildboar Spain	Unknown	Cultured	Not available
*M. tuberculosis* (4)					
15‐961‐2	*M. tuberculosis*	Elephant 1	Lung	Cultured	Clinical specimen
15‐961‐1	*M. tuberculosis*	Elephant 1	Pharyngeal swab	Not cultivated	Clinical specimen
15‐1115‐2	*M. tuberculosis*	Elephant 2	Lung	Cultured	Not available
15‐1221‐1	*M. tuberculosis*	Elephant 3	Lung	Cultured	Not available

**Table A2:** Exclusivity panel consisting of 41 nontuberculous mycobacteria and 3 nonmycobacterial species for specificity testing of the high‐resolution melting (HRM) assays 2 and 3

Species	No. of isolates	Species	No. of isolates
*M. abscessus* sp.	2	*M. malmoense*	1
*M. avium* subsp. *avium*	2	*M. marinum*	1
*M. avium* subsp*. hominissuis*	32	*M. monacense*	3
*M. avium* subsp. *paratuberculosis*	1	*M. nebraskense*	1
*M. avium* subsp. *silvaticum*	1	*M. neoaurum*	5
*M. bourgelatii*	1	*M. nonchromogenicum*	7
*M. celatum*	1	*M. palustre*	1
*M. chelonae* subsp. *chelonae*	1	*M. parafortuitum*	2
*M. chimaera*/*intracellulare*/*youngonense*	1	*M. paragordonae*	6
*M. chitae*	1	*M. peregrinum*	2
*M. elephantis*	1	*M. persicum*	2
*M. engbaekii*	1	*M. phlei*	3
*M. europaeum*	1	*M. scrofulaceum*	1
*M. fortuitum*/*porcinum*	1	*M. simiae*	1
*M. goodii*	1	*M. smegmatis*	1
*M. gordonae*	2	*M. szulgai*	1
*M. hassiacum*	1	*M. terrae*	1
*M. interjectum*/*paraense*	1	*M. vaccae*	4
*M. intermedium*	1	*M. xenopi*	5
*M. intracellulare*	3	*Nocardia paucivorans*	1
*M. kansasii*	10	*Escherichia coli*	1
*M. lymphaticum*	1	*Streptococcus suis*	1

**Table A3:** Raw data set and statistical parameters generated from the intra‐ and interassay variability of high‐resolution melting (HRM) assay 2 using a randomly chosen subset of 22 cultured samples

Isolate			Run 1						Run 2						Run 3						Inter‐Assay			
MTBC member	Sample no	Origin	Ct	*T_m_*	Mean *T_m_*	*SD*	CV%	Intra‐assay CV%	Ct	*T_m_*	Mean *T_m_*	*SD*	CV%	Intra‐assay CV%	Ct	*T_m_*	Mean *T_m_*	*SD*	CV%	Intra‐assay CV%	Mean *T_m_*	*SD*	CV%	Inter‐assay CV%
*M. microti*	ATCC 19422		20.65	83.13					20.87	83.25					19.74	83.28					83.22	0.08	0.10	
*M. bovis* BCG Pasteur	ATCC 35734		24.67	82.62					24.82	82.77					23.57	82.70					82.70	0.08	0.09	
*M. tuberculosis*	H37RV		22.41	83.55					22.44	83.75					20.97	83.60					83.63	0.10	0.12	
*M. tuberculosis*	15‐961 2B	Elephant	14.87	83.60	83.58	0.02	0.02		15.11	83.80	83.80	0.00	0.00		14.01	83.70	83.69	0.02	0.02		83.69	0.11	0.13	
			14.95	83.58					15.13	83.80					14.05	83.70								
			14.96	83.57					15.14	83.80					13.94	83.67								
*M. tuberculosis*	15‐1115‐2	Elephant	16.34	83.55	83.54	0.01	0.01		16.69	83.87	83.86	0.03	0.03		15.32	83.63	83.65	0.03	0.03		83.69	0.16	0.19	
			16.71	83.55					16.65	83.83					15.47	83.65								
			16.73	83.53					16.75	83.88					15.46	83.68								
*M. tuberculosis*	15‐1221‐1	Elephant	19.59	83.53	83.53	0.01	0.01	0.01	19.98	83.87	83.83	0.04	0.04	0.02	18.45	83.60	83.67	0.06	0.07	0.04	83.68	0.15	0.18	0.17
			19.80	83.52					20.02	83.83					18.45	83.68								
			19.77	83.53					19.75	83.80					18.47	83.72								
*M. caprae*	229171	Cow	20.74	83.05	83.05	0.00	0.00		21.20	83.23	83.24	0.01	0.01		19.79	83.23	83.23	0.02	0.02		83.17	0.11	0.13	
			37.30						21.09	83.25					19.76	83.22								
			21.07	83.05					21.28	83.23					19.55	83.25								
*M. caprae*	22914	Cow	20.60	83.10	83.12	0.03	0.03		20.97	83.30	83.26	0.04	0.04		19.52	83.27	83.25	0.02	0.02		83.21	0.08	0.10	
			20.68	83.10					21.02	83.25					19.59	83.25								
			20.71	83.15					20.79	83.23					19.48	83.23								
*M. caprae*	22848	Cow	22.93	83.07	83.04	0.02	0.03		23.24	83.20	83.19	0.01	0.01		21.68	83.23	83.20	0.03	0.04		83.14	0.09	0.10	
			22.94	83.03					23.20	83.18					21.77	83.20								
			22.90	83.03					23.34	83.18					21.53	83.17								
*M. caprae*	22966	Cow	19.89	83.13	83.15	0.02	0.02		20.04	83.35	83.33	0.04	0.05		18.60	83.25	83.26	0.02	0.02		83.25	0.09	0.11	
			19.84	83.15					19.97	83.35					18.46	83.25								
			19.81	83.17					20.04	83.28					18.84	83.28								
*M. caprae*	13‐450	Cow	18.04	83.20	83.16	0.04	0.04		18.23	83.30	83.31	0.02	0.02		17.00	83.28	83.28	0.01	0.01		83.25	0.08	0.09	
			18.04	83.13					18.10	83.33					16.99	83.28								
			18.13	83.15					18.21	83.30					16.93	83.27								
*M. caprae*	13‐162	Cow	22.08	83.13	83.10	0.03	0.03	0.03	22.43	83.23	83.24	0.01	0.01	0.03	20.91	83.25	83.26	0.01	0.01	0.02	83.20	0.08	0.10	0.11
			21.93	83.10					22.23	83.25					20.75	83.25								
			22.01	83.08					22.33	83.23					20.87	83.27								
*M. bovis*	20593	Cow	26.50	82.65	82.64	0.01	0.01		27.13	82.88	82.93	0.06	0.08		25.69	82.68	82.69	0.01	0.01		82.75	0.15	0.18	
			24.89	82.65					23.85	83.00					24.93	82.70								
			25.63	82.63					24.38	82.90					25.46	82.70								
*M. bovis*	20594	Cow	25.56	82.60	82.61	0.02	0.02		26.03	82.88	82.88	0.03	0.03		24.80	82.72	82.71	0.02	0.03		82.73	0.14	0.16	
			24.81	82.60					25.75	82.85					24.77	82.72								
			25.43	82.63					25.79	82.90					23.95	82.68								
*M. bovis*	20175	Cow	18.77	82.80	82.74	0.05	0.06		18.90	82.98	82.93	0.07	0.09		17.68	82.80	82.78	0.03	0.03		82.82	0.10	0.12	
			18.80	82.70					18.92	82.97					17.53	82.80								
			18.81	82.73					18.94	82.85					17.66	82.75								
*M. bovis*	20596	Cow	21.91	82.67	82.67	0.08	0.09		22.37	82.95	82.94	0.01	0.01		21.14	82.80	82.76	0.04	0.04		82.79	0.14	0.17	
			22.42	82.75					22.47	82.93					20.81	82.75								
			22.56	82.60					22.43	82.95					21.01	82.73								
*M. bovis*	22667	Cow	19.91	82.80	82.80	0.00	0.00		20.04	82.93	82.92	0.01	0.01		18.95	82.78	82.78	0.01	0.01		82.83	0.08	0.10	
			19.84	82.80					20.05	82.92					18.79	82.77								
			19.81	82.80					20.05	82.92					19.09	82.78								
*M. bovis*	22539	Cow	21.42	82.73	82.76	0.04	0.04	0.04	21.80	82.92	82.92	0.00	0.00	0.04	20.49	82.77	82.76	0.01	0.01	0.02	82.81	0.09	0.11	0.14
			21.55	82.75					21.44	82.92					20.44	82.75								
			21.38	82.80					21.60	82.92					20.52	82.75								
*M. microti*	22928	Cat	16.90	83.17	83.17	0.02	0.02		17.13	83.40	83.42	0.02	0.02		16.01	83.30	83.31	0.01	0.01		83.30	0.13	0.15	
			16.93	83.18					17.13	83.43					15.89	83.30								
			17.01	83.15					17.08	83.43					16.13	83.32								
*M. microti*	15‐1765	Cat	21.77	83.18	83.16	0.03	0.03		22.21	83.32	83.30	0.02	0.02		20.79	83.28	83.28	0.02	0.02		83.25	0.08	0.09	
			21.44	83.17					21.93	83.30					19.99	83.27								
			21.78	83.13					22.05	83.28					20.59	83.30								
*M. microti*	14‐58	Cat	24.40	83.02	83.02	0.01	0.01		24.39	83.28	83.27	0.02	0.02		23.44	83.28	83.28	0.02	0.02		83.19	0.15	0.18	
			24.39	83.02					24.64	83.28					23.18	83.30								
			24.31	83.03					24.43	83.25					23.45	83.27								
*M. microti*	14‐690	Alpaca	15.10	83.05	83.05	0.03	0.03		15.33	83.30	83.29	0.01	0.01		14.28	83.30	83.29	0.01	0.01		83.21	0.14	0.17	
			15.13	83.07					15.27	83.28					14.20	83.30								
			15.21	83.02					15.29	83.30					14.24	83.28								
*M. microti*	16‐2156	Cat	23.07	83.07	83.08	0.02	0.02		23.48	83.30	83.31	0.01	0.01		22.32	83.27	83.27	0.00	0.00		83.22	0.12	0.14	
			23.49	83.08					23.60	83.30					22.17	83.27								
			23.49	83.10					23.57	83.32					22.01	83.27								
*M. microti*	1522744	Cat	22.79	83.12	83.09	0.03	0.03		22.89	83.33	83.29	0.03	0.04		21.82	83.27	83.26	0.01	0.01		83.21	0.11	0.13	
			22.67	83.07					22.97	83.27					21.74	83.27								
			22.68	83.08					22.94	83.27					21.49	83.25								
*M. microti*	16‐1347	Cat	18.92	83.13	83.15	0.03	0.03	0.02	19.20	83.30	83.27	0.03	0.03	0.02	17.52	83.30	83.30	0.00	0.00	0.01	83.24	0.08	0.10	0.14
			18.94	83.13					18.99	83.25					17.21	83.30								
			18.87	83.18					18.86	83.25					17.32	83.30								

**Table A4:** Raw data set and statistical parameters generated from the intra‐ and interassay variability of high‐resolution melting (HRM) assay 3 using a randomly chosen subset of 9 cultured samples and 7 clinical specimens

Isolate			Run 1						Run 2						Run 3						Inter‐Assay			
MTBC member	Sample no	Origin	Ct	*T_m_*	Mean *T_m_*	*SD*	CV%	Intra‐assay CV%	Ct	*T_m_*	Mean *T_m_*	*SD*	CV%	Intra‐assay CV%	Ct	*T_m_*	Mean *T_m_*	*SD*	CV%	Intra‐assay CV%	Mean *T_m_*	*SD*	CV%	Inter‐assay CV%
*M. microti*	ATCC 19422		26.37	86.83					28.03	86.83					28.56	86.83					86.83	0.00	0.00	
*M. tuberculosis*	H37RV		29.35	86.82					29.36	86.82					29.03	86.85					86.83	0.02	0.02	
*M. bovis* isolate	20593	Cow	35.04	86.88	86.86	0.02	0.02		33.95	86.85	86.85	0.00	0.00		33.73	86.87	86.90	0.03	0.03		86.87	0.02	0.03	
			35.10	86.85					33.59	86.85					33.52	86.92								
			35.36	86.85					33.70	86.85					32.61	86.90								
*M. bovis* isolate	20594	Cow	32.92	86.85	86.85	0.00	0.00		32.73	86.85	86.86	0.02	0.02		32.36	86.83	86.88	0.05	0.05		86.86	0.02	0.02	
			33.48	86.85					32.56	86.85					32.62	86.90								
			33.28	86.85					32.83	86.88					32.60	86.92								
*M. bovis* isolate	20609	Cow	35.18	86.83	86.83	0.02	0.02		34.71	86.87	86.87	0.03	0.03		33.73	86.90	86.87	0.06	0.07		86.86	0.02	0.02	
			34.74	86.85					33.89	86.85					33.87	86.80								
			34.54	86.82					34.39	86.90					33.60	86.90								
*M. bovis* isolate	20608	Cow	28.92	86.82	86.82	0.01	0.01		28.69	86.83	86.84	0.01	0.01		27.96	86.78	86.77	0.04	0.04		86.81	0.04	0.04	
			28.55	86.83					28.50	86.85					28.36	86.73								
			29.49	86.82					28.75	86.85					28.46	86.80								
*M. bovis* isolate	20596	Cow	29.93	86.78	86.79	0.01	0.01		29.35	86.83	86.84	0.01	0.01		29.37	86.80	86.80	0.00	0.00		86.81	0.02	0.03	
			29.69	86.80					29.25	86.85					28.64	86.80								
			29.87	86.80					29.30	86.83					28.92	86.80								
*M. bovis* isolate	20600	Cow	27.83	86.83	86.83	0.00	0.00		29.51	86.85	86.83	0.02	0.02		29.82	86.85	86.84	0.01	0.01		86.84	0.01	0.01	
			29.66	86.83					30.07	86.83					29.91	86.83								
			30.09	86.83					30.49	86.82					29.91	86.85								
*M. bovis* isolate	20606	Cow	28.36	86.80	86.81	0.02	0.02	0.01	27.94	86.80	86.78	0.03	0.03	0.02	27.42	86.83	86.84	0.01	0.01	0.03	86.81	0.03	0.03	0.03
			27.86	86.80					28.01	86.78					27.34	86.85								
			28.12	86.83					27.88	86.75					27.47	86.83								
*M. bovis* specimen	20593	Cow	19.04	86.83	86.84	0.01	0.01		18.82	86.82	86.80	0.02	0.02		18.64	86.88	86.92	0.04	0.04		86.85	0.06	0.07	
			18.84	86.85					19.19	86.80					18.80	86.92								
			18.65	86.85					18.95	86.78					18.68	86.95								
*M. bovis* specimen	20594	Cow	36.86		86.88	0.01	0.01		16.22	86.78	86.78	0.02	0.02		16.16	86.90	86.91	0.01	0.01		86.86	0.06	0.07	
			16.18	86.88					16.51	86.80					16.18	86.92								
			16.75	86.87					16.55	86.77					16.07	86.90								
*M. bovis* specimen	20609	Cow	29.46	86.85	86.85	0.00	0.00		28.99	86.80	86.82	0.03	0.03		28.66	86.88	86.85	0.03	0.03		86.84	0.02	0.02	
			29.75	86.85					29.35	86.80					28.51	86.85								
			29.04	86.85					29.45	86.85					28.57	86.83								
*M. bovis* specimen	20608	Cow	17.18	86.85	86.88	0.03	0.03		17.34	86.85	86.86	0.01	0.01		17.03	86.90	86.88	0.03	0.03		86.87	0.01	0.02	
			17.43	86.90					17.60	86.85					17.13	86.90								
			17.14	86.88					17.64	86.87					17.22	86.85								
*M. bovis* specimen	20596	Cow	16.60	86.85	86.86	0.04	0.05		16.99	86.90	86.90	0.00	0.00		16.66	86.87	86.86	0.01	0.01		86.87	0.03	0.03	
			17.02	86.90					17.01	86.90					16.54	86.85								
			16.84	86.82					17.14	86.90					16.42	86.85								
*M. bovis* specimen	20600	Cow	16.88	86.85	86.85	0.00	0.00		17.15	86.88	86.88	0.03	0.03		16.97	86.85	86.84	0.01	0.01		86.86	0.02	0.02	
			16.83	86.85					17.47	86.90					16.96	86.85								
			16.74	86.85					17.24	86.85					16.64	86.83								
*M. bovis* specimen	20606	Cow	17.22	86.85	86.85	0.00	0.00	0.01	17.18	86.85	86.85	0.02	0.02	0.02	16.72	86.85	86.83	0.03	0.03	0.02	86.84	0.01	0.02	0.04
			17.34	86.85					17.31	86.83					16.77	86.80								
			16.90	86.85					17.23	86.87					16.87	86.83								
*M. bovis* BCG Tice isolate	ATCC 27289		30.43	86.40	86.37	0.04	0.04		30.70	86.40	86.40	0.02	0.02		30.26	86.43	86.44	0.01	0.01		86.40	0.04	0.04	
			30.84	86.38					30.51	86.38					30.14	86.45								
			30.73	86.33					30.22	86.42					29.81	86.45								
*M. bovis* BCG Pasteur isolate	ATCC 35734		30.62	86.45	86.44	0.02	0.02	0.03	31.32	86.42	86.41	0.03	0.03	0.03	30.56	86.47	86.46	0.03	0.04	0.03	86.44	0.02	0.03	0.04
			30.72	86.42					31.15	86.38					30.23	86.42								
			30.78	86.45					31.14	86.43					30.27	86.48								

**Table A5:** Raw data set and statistical parameters generated from the intra‐ and interassay variability of high‐resolution melting (HRM) assay 2 using a randomly chosen subset of 18 clinical specimens

Isolate			Run 1						Run 2						Run 3						Inter‐Assay			
MTBC member	Sample no	Origin	Ct	*T_m_*	Mean *T_m_*	*SD*	CV%	Intra‐assay CV%	Ct	*T_m_*	Mean *T_m_*	*SD*	CV%	Intra‐assay CV%	Ct	*T_m_*	Mean *T_m_*	*SD*	CV%	Intra‐assay CV%	Mean *T_m_*	*SD*	CV%	Inter‐assay CV%
*M. microti*	ATCC 19422		21.28	83.30					21.82	83.25					21.25	83.08					83.21	0.12	0.14	
*M. bovis* BCG Pasteur	ATCC 35734		25.78	82.82					25.33	82.73					25.23	82.62					82.72	0.10	0.12	
*M. tuberculosis*	H37RV		22.87	83.85					22.65	83.67					22.62	83.58					83.70	0.14	0.16	
*M. tuberculosis*	15‐961 2A	Elephant	29.98	83.58	83.59	0.01	0.01		29.48	83.50	83.53	0.03	0.03		29.16	83.47	83.44	0.02	0.03		83.52	0.08	0.09	
			29.90	83.60					29.72	83.55					29.33	83.43								
			30.23	83.60					29.32	83.53					29.19	83.43								
*M. tuberculosis*	19‐691 1A + B	Elephant	24.51	83.77	83.81	0.03	0.04	0.03	24.78	83.68	83.68	0.03	0.03	0.03	24.44	83.60	83.57	0.03	0.03	0.03	83.68	0.12	0.14	0.12
			24.84	83.82					24.64	83.70					24.49	83.55								
			24.63	83.83					24.78	83.65					24.78	83.55								
*Microsoft. caprae*	22971	Cow	31.58	83.20	83.22	0.02	0.02		31.45	83.13	83.13	0.02	0.02		31.21	83.08	82.99	0.08	0.09		83.11	0.11	0.14	
			31.37	83.23					30.75	83.12					31.27	82.95								
			31.39	83.22					30.96	83.15					31.04	82.95								
*M. caprae*	22914	Cow	29.37	83.17	83.16	0.01	0.01		28.86	83.00	83.02	0.03	0.03		28.57	82.88	82.88	0.02	0.02		83.02	0.14	0.17	
			29.04	83.15					28.59	83.05					28.93	82.87								
			28.91	83.17					28.54	83.00					28.40	82.90								
*M. caprae*	22848	Cow	19.00	83.35	83.34	0.04	0.04		19.60	83.25	83.26	0.01	0.01		19.69	83.08	83.07	0.01	0.01		83.22	0.14	0.16	
			19.33	83.37					19.27	83.27					19.58	83.07								
			19.85	83.30					19.16	83.25					19.57	83.07								
*M. caprae*	13‐162	Cow	32.47	83.10	83.11	0.02	0.02	0.02	31.56	82.98	82.98	0.03	0.03	0.02	31.10	82.87	82.87	0.02	0.02	0.03	82.98	0.12	0.15	0.15
			33.03	83.10					31.89	82.95					32.23	82.85								
			31.85	83.13					31.93	83.00					31.75	82.88								
*M. bovis*	20593	Cow	14.57	82.90	82.98	0.07	0.08		14.31	82.80	82.79	0.01	0.01		14.18	82.68	82.69	0.01	0.01		82.82	0.15	0.18	
			14.67	83.02					14.32	82.80					14.27	82.70								
			14.58	83.02					14.23	82.78					14.81	82.70								
*M. bovis*	20594	Cow	12.65	83.07	83.02	0.04	0.05		12.54	82.85	82.85	0.03	0.03		11.91	82.80	82.78	0.02	0.02		82.89	0.12	0.15	
			12.60	83.00					12.86	82.83					11.48	82.77								
			12.49	83.00					12.58	82.88					11.69	82.77								
*M. bovis*	20609	Cow	22.46	82.87	82.89	0.03	0.04		22.84	82.78	82.76	0.02	0.02		22.50	82.65	82.63	0.03	0.03		82.76	0.13	0.16	
			22.49	82.93					22.65	82.75					22.62	82.65								
			22.29	82.88					22.45	82.75					22.55	82.60								
*M. bovis*	20608	Cow	13.54	83.03	83.03	0.02	0.02		13.30	82.85	82.84	0.01	0.01		13.11	82.68	82.70	0.02	0.02		82.86	0.17	0.20	
			13.19	83.02					13.46	82.85					13.29	82.72								
			13.31	83.05					13.16	82.83					13.13	82.70								
*M. bovis*	20596	Cow	12.86	83.02	83.05	0.03	0.03		12.81	82.83	82.83	0.00	0.00		13.02	82.65	82.68	0.04	0.04		82.85	0.18	0.22	
			12.93	83.05					12.82	82.83					12.97	82.68								
			13.10	83.07					12.84	82.83					12.99	82.72								
*M. bovis*	20600	Cow	13.19	83.00	83.00	0.00	0.00	0.04	12.90	82.82	82.83	0.02	0.02	0.02	13.18	82.65	82.66	0.04	0.05	0.03	82.83	0.17	0.21	0.19
			13.17	83.00					13.08	82.82					12.77	82.62								
			13.07	83.00					13.06	82.85					12.95	82.70								
*M. microti*	22928	Cat	21.04	83.28	83.32	0.04	0.04		21.00	83.22	83.22	0.01	0.01		20.82	83.03	83.04	0.01	0.01		83.20	0.14	0.17	
			21.25	83.33					21.16	83.22					20.88	83.05								
			21.00	83.35					20.93	83.23					20.82	83.05								
*M. microti*	15‐342	Alpaca	15.26	83.35	83.36	0.01	0.01		14.98	83.27	83.24	0.02	0.03		15.10	83.03	83.06	0.02	0.03		83.22	0.15	0.18	
			15.15	83.35					14.60	83.23					14.91	83.07								
			15.19	83.37					15.04	83.23					15.15	83.07								
*M. microti*	17‐1084	Cat	22.93	83.27	83.29	0.02	0.02		22.99	83.20	83.19	0.02	0.02		23.08	83.05	83.07	0.03	0.03		83.18	0.11	0.13	
			22.82	83.30					22.95	83.20					22.87	83.05								
			22.90	83.30					23.20	83.17					23.15	83.10								
*M. microti*	15‐1955	Cat	25.48	83.23	83.23	0.02	0.02		25.79	83.10	83.12	0.04	0.05		26.19	83.00	82.99	0.04	0.04		83.12	0.12	0.15	
			25.21	83.22					25.61	83.10					25.74	82.95								
			25.37	83.25					25.65	83.17					26.35	83.02								
*M. microti*	14‐690	Alpaca	20.03	83.32	83.32	0.00	0.00		20.00	83.20	83.22	0.02	0.02		20.13	83.05	83.03	0.03	0.03		83.19	0.15	0.17	
			19.84	83.32					19.90	83.23					19.94	83.05								
			19.90	83.32					20.01	83.23					19.98	83.00								
*M. microti*	17‐549	Llama	30.79	83.22	83.23	0.04	0.05	0.02	30.07	83.10	83.13	0.03	0.03	0.03	30.24	82.90	82.96	0.05	0.06	0.04	83.11	0.14	0.17	0.16
			30.23	83.20					30.62	83.15					30.03	83.00								
			30.78	83.28					30.20	83.13					29.89	82.98								
